# Non-Invasive Detection of Nasopharyngeal Carcinoma Using Volatile Organic Compounds

**DOI:** 10.3390/metabo15090632

**Published:** 2025-09-22

**Authors:** Chuan Hao Gui, Zhunan Jia, Alex Chengyao Tham, Khai Beng Chong, Zihao Xing, Fuchang Zhang, Fang Du, Yaw Khian Chong, Hao Li, Ernest Weizhong Fu, Jereme Yijin Gan, Agnes Si Qi Chew, Ming Yann Lim

**Affiliations:** 1 Tan Tock Seng Hospital, Singapore 308433, Singapore; 2 Breathonix Pte Ltd., Singapore 118258, Singapore

**Keywords:** nasopharyngeal carcinoma, volatile organic compounds, breath analysis

## Abstract

**Background:** Nasopharyngeal carcinoma (NPC) is a leading head and neck cancer in Asia, where late-stage presentation contributes to poor survival. Non-invasive diagnostic strategies such as breath analysis may improve early detection. **Objectives:** This study aimed to investigate whether volatile organic compound (VOC) features in exhaled breath, detected using proton transfer reaction mass spectrometry (PTR-MS), can distinguish NPC patients from healthy controls. **Methods:** Breath samples were collected from 50 NPC patients and 40 healthy controls. PTR-TOF-MS was used to measure exhaled VOC features. Group comparisons were performed using univariate analysis, while multivariable regression was adjusted for age, sex, BMI, smoking, and medication use. Multivariate methods, including principal component analysis (PCA) and random forest classification, were used to assess discriminatory potential. **Results:** Seven distinct VOC features (measured as *m*/*z* values) showed significant differences between NPC patients and healthy controls, with m089 and m175 emerging as the strongest markers of distinction. PCA after normalization revealed clearer separation between NPC patients and controls. Random forest models incorporating significant VOCs achieved moderate classification accuracy, and the results remained robust after adjusting for confounders. **Conclusions:** PTR-MS breath analysis can detect disease-specific VOC features in NPC and shows promise as a non-invasive diagnostic tool. Larger validation studies and definitive compound identification are needed to confirm clinical utility.

## 1. Introduction

Nasopharyngeal carcinoma (NPC) is a type of head and neck cancer that originates from the epithelial cells lining the nasopharynx, a region anatomically close to the upper airway and nasal passages [1,2,3,4,5]. NPC is particularly notable for its association with Epstein–Barr virus (EBV) infection, genetic predispositions, and environmental factors such as dietary habits and exposure to carcinogens [6]. Clinical features of NPC include nonspecific symptoms like nasal congestion, epistaxis, hearing loss, and cervical lymphadenopathy [7,8,9]. Early detection and accurate staging are critical for NPC management, as the disease can metastasize to regional lymph nodes and distant organs.

Currently, there are no widely available non-invasive screening tests for NPC. The primary screening method, EBV serology, while helpful, has limitations in terms of sensitivity and specificity. Moreover, nasoendoscopy, the gold standard for NPC detection, is not easily accessible outside of specialist settings, which complicates early detection efforts. The lack of effective non-invasive tests underscores a critical gap in NPC diagnosis and screening.

Accurate diagnosis of NPC involves a multidisciplinary approach. This typically includes nasopharyngoscopy for visual examination of the nasopharynx, imaging modalities such as magnetic resonance imaging (MRI) and computed tomography (CT) scans for tumor localization and staging, and biopsy for histological confirmation of malignancy [10,11,12]. Molecular and genetic tests may also play a role in identifying specific biomarkers and prognostic indicators in NPC patients.

However, the diagnostic workup for NPC, including nasopharyngoscopy, imaging studies, and biopsy, can be invasive, time-consuming, and costly. Imaging modalities like MRI and CT scans expose patients to ionizing radiation and require skilled interpretation for accurate tumor localization and staging. Biopsy procedures carry risks of bleeding, infection, and sampling errors, necessitating careful consideration of clinical indications and the potential benefits versus harms.

Volatile organic compounds (VOCs) are a diverse group of organic chemicals present in exhaled breath, originating from various metabolic processes within the body [13,14]. Detected in trace amounts, these compounds can serve as potential biomarkers for a wide range of diseases, including nasopharyngeal carcinoma (NPC). Biomarkers are measurable indicators of a biological condition, and in the context of breath analysis, they offer a non-invasive method for disease detection and monitoring. Multiple studies have demonstrated differences in breath VOCs between patients with and without cancer. A recent systematic review [15] summarized more than a hundred candidate VOCs across cancer studies, with commonly reported examples including acetone, 2-butanone, isoprene, nonanal, and tetradecane, which have been repeatedly linked to altered metabolism in cancer patients.

Although breath VOC analysis has not yet been widely applied to NPC, previous studies on head and neck squamous cell carcinoma [16] and other cancers [15] have consistently demonstrated the value of VOCs as biomarkers of tumor-associated metabolic changes. These findings support the biological plausibility of our approach and provide a strong rationale for extending this line of research to NPC. To date, no study has specifically examined breath analysis in NPC, despite it being one of the most prevalent head and neck cancers in Asian populations [17].

Breath tests are a compelling diagnostic approach because they are non-invasive and easy to administer and allow for rapid analysis [18]. The process involves collecting exhaled breath from patients, which is then analyzed using advanced techniques such as proton transfer reaction mass spectrometry (PTR-MS). PTR-MS is a highly sensitive method that enables real-time detection and quantification of VOCs in breath samples, making it a valuable tool for identifying disease-specific biomarkers.

Breath analysis using PTR-MS and the detection of VOC features represent a promising frontier in medical diagnostics. This approach not only improves our understanding of the metabolic changes associated with disease but also creates opportunities for personalized medicine, where treatments may be tailored to the individual patient’s VOC profile.

## 2. Materials and Methods

### 2.1. Study Population

The study involved a total of 90 participants, divided into three distinct groups: 50 patients with nasopharyngeal carcinoma (NPC) and 40 healthy controls. All participants were recruited from Tan Tock Seng Hospital Singapore, a major healthcare institution renowned for its comprehensive medical services and research facilities, between May 2022 and June 2024, after obtaining informed consent. The difference in group sizes reflects the practical constraints of recruitment during the study period, with all eligible NPC patients enrolled consecutively and healthy controls recruited on a voluntary basis. Although the groups were modestly unequal in size, both provided adequate statistical power, and confounder-adjusted analyses were subsequently applied to reduce potential bias.

Participants in the NPC group were recruited following a confirmed diagnosis of NPC from post-nasal space biopsy results. The control group participants were voluntarily recruited after undergoing nasoendoscopy, which revealed no significant abnormalities and did not meet any of the exclusion criteria outlined below. The exclusion criteria for this study included recent food or beverage consumption within the past hour or adherence to a ketogenic diet, alcohol intake within the past 6 h, a history of cancer, pregnancy, respiratory or heart failure, liver dysfunction or failure, renal failure, and uncontrolled diabetes mellitus. These exclusion criteria were selected based on the proposed framework for conducting and reporting studies on VOCs, with the aim of minimizing confounding factors [19], particularly those affecting metabolic processes or respiratory function, which could interfere with the detection of volatile organic compounds. Other relevant demographic data, including age, gender, body mass index (BMI), smoking status, medical history, and long-term medication use, were also collected for this study. NPC stages were determined based on tumor characteristics, nodal spread, and distant metastasis, in accordance with the 8th Edition of the American Joint Committee on Cancer (AJCC) Staging System for Head and Neck Cancer [20].

The trial was approved by the NHG Domain Specific Review Board (DSRB) (Ref: 2019/00345), and all enrolled subjects gave signed informed consent prior to the study.

### 2.2. Breath Collection, PTR-TOF-MS Measurement, and Analysis

Breath samples were collected from both NPC patients and healthy controls using sterile Tedlar bags in the same clinic room to minimize the influence of ambient air. The participants were instructed to provide three consecutive end-tidal exhalations under study team supervision to ensure the capture of alveolar air. The bags were sealed immediately after collection and transported to the research laboratory on the same day for PTR-MS analysis. This standardized procedure minimized environmental contamination and ensured comparability across the groups.

The breath samples were measured using a PTR-MS TOF1000 (Ionicon Analytik GmbH, Innsbruck, Austria), with technical specifications provided in Table 1. PTR-MS consists of an ionization section and a detection section [21]. During the ionization process, protonated water ions (H_3_O^+^) were generated through a hollow cathode discharge in the ion source. These H_3_O^+^ ions were subsequently introduced into the drift tube by an electric drift field, facilitating the chemical ionization of volatile organic compounds (VOCs) in breath samples via proton transfer reactions (PTR). Only VOCs with a higher proton affinity (PA) value than that of H_2_O molecules underwent ionization by H_3_O^+^ and proceeded to the detection section. The ionized VOCs were then directed by an electric field towards the time-of-flight mass spectrometer (TOF-MS), where they were differentiated and detected based on their mass-to-charge ratio (*m*/*z*). Because of this ionization method, its molecular weight after ionization is one proton greater than its molecular weight before ionization. Quantification of common VOCs was performed using calibration gas cylinders containing known concentrations of reference compounds. By measuring the count rate of both reagent ions and product ions, real-time quantification is achieved. PTR-MS can detect compounds at parts per billion by volume or even parts per trillion levels in real time.

Key operational parameters of the PTR-MS instrument included a drift tube voltage of 600 V, a temperature of 80 °C, a drift tube pressure of 2.3 mbar, and an E/N ratio of 139 Td. Additionally, the sampling line and buffer tube were maintained at 70 °C. The sampling line and buffer tube were maintained at a temperature of at least 70 °C to minimize the condensation of water vapor and reduce the risk of contamination by viruses and other particulate matter. Elevated temperatures help to prevent the accumulation of moisture, which can act as a carrier for pathogens, thus improving the accuracy and safety of the sample collection process.

After the participants registered their personal data and signed consent forms, they were instructed by a study team member to exhale into a Tedlar bag, which was then sealed and transported to the research laboratory on the same day. Before the measurements, the device was calibrated. The Tedlar bag was connected to the PTR-MS device and sealed using laboratory film (Parafilm). Initially, the PTR-MS was connected to the external environment until the pressure parameters stabilized. Once stable, the valve of the Tedlar bag was opened, and the device began drawing air from the bag. Observing the pressure parameters ensured that they were stable before data collection commenced. This procedure minimizes pressure fluctuations in the drift tube, ensuring accurate readings [22,23].

The procedure described addresses several potential issues and streamlines the collection process. Starting measurements from normal atmospheric pressure avoids the lengthy stabilization period required if starting from lower pressures, which can sometimes result in the device reaching only half the target pressure and significantly skewing the data. Consequently, we avoided switching from standby mode to active measurement mode. Since the Tedlar bag valve is initially closed, if the bag is connected from the start, the lack of gas input prevents the PTR-MS pressure from rising until the valve is opened. This situation is akin to starting from standby mode, which we aim to avoid.

The PTR-MS calculates the concentration of all VOCs and saves the data in .h5 files [21]. This raw concentration data was processed using Viewer software 4.2.0 (Ionicon Analytik GmbH, Innsbruck, Austria) for mass calibration and peak data calculation. The software identified background air and exhaled breath, selecting and averaging data points from the end-tidal phase for the three exhalations from each subject. VOCs with concentrations lower in breath than in the background were excluded from the subsequent data analysis. An Excel file containing the list of VOCs and their concentrations (in parts per billion, ppb) was generated for each sample.

### 2.3. Statistical Analysis

All statistical analyses were conducted using R (version 4.2.2; R Foundation for Statistical Computing, Vienna, Austria). Continuous variables (age, BMI) were summarized as mean ± standard deviation and compared between the groups using the Wilcoxon rank-sum test. Categorical variables (gender, smoking status, medication use) were presented as counts and percentages and compared using chi-squared tests. Standardized mean differences (SMDs) were calculated to assess group balance.

Significance testing of VOCs between the NPC patients and controls was performed using the Wilcoxon rank-sum test, with *p* < 0.05 considered statistically significant. To evaluate confounder effects, multivariate analysis of variance (MANOVA) was applied, and confounder-adjusted principal component analysis (PCA) was conducted using residualized data. Model performance was assessed using random forest classifiers, with receiver operating characteristic (ROC) curve and area under the curve (AUC) values generated to evaluate predictive accuracy.

## 3. Results

Data analysis was performed using R. Multiple dimensions of the data were analyzed, and significance analysis was performed to rank *m*/*z* values based on their *p*-values to identify potential biomarkers. The data were also visualized using principal component analysis (PCA) plots, heatmaps, and correlation maps.

### 3.1. Study Characteristics

Table 2 shows the demographics comparing the NPC and control groups. The NPC group consisted of 50 individuals with a mean age of 58.98 ± 11.38 years, while the control group included 40 individuals with a mean age of 43.38 ± 12.70 years. At the time of diagnosis and recruitment for breath analysis, 6% of the participants in the NPC group were classified as Stage I, 16% as Stage II, 36% as Stage III, and 42% as Stage IV.

### 3.2. NPC vs. Control Analysis

#### 3.2.1. Significance Analysis

In the comparison between NPC patients and control subjects, several VOCs were identified as significant biomarkers. To evaluate the differences in VOC levels between the two groups, the Wilcoxon rank-sum test (also known as the Mann–Whitney U test) was employed. This non-parametric statistical method is particularly suited for small sample sizes or data that do not follow a normal distribution, making it a robust choice for analyzing the metabolic data in this study. Table 3 lists the biomarkers and their *p*-values, with m088, m088.070, m089, m090, m097.071 cycloheptene, m098, and m175 showing significant differences. The most notable markers were m089 and m175, with *p*-values of 0.00656763 and 0.001683542, respectively, indicating their potential as robust biomarkers for NPC.

#### 3.2.2. Assessment of Baseline Characteristics and Group Balance

To investigate the potential impact of confounding factors on group assignment, we analyzed the baseline characteristics of the NPC and healthy control groups (including age, sex, BMI, long-term medication use, and smoking status) and assessed the balance of these variables between the two groups. Baseline characteristics between the NPC and healthy control groups were compared using the CreateTableOne function from the tableone package in R. Continuous variables (e.g., age, BMI) were summarized as mean ± standard deviation, and categorical variables (e.g., gender, medication status, smoking status) were presented as counts and percentages. Standardized mean differences (SMDs) were calculated for each variable to assess the balance of baseline characteristics between the groups. All variables were analyzed at all categorical levels to provide a complete comparison.

The baseline characteristics analysis as shown in Table 4 revealed significant imbalances between the NPC patients and healthy controls in gender distribution (*p* = 0.010, SMD = 0.612) and smoking status (*p* = 0.007, SMD = 0.703), with the NPC group showing higher proportions of males (86.0% vs. 60.0%) and current/former smokers (68.0% vs. 35.0%). While age showed moderate imbalance (SMD = 0.335), BMI and medication use were well-balanced between the groups (SMD < 0.1).

**Figure 1:**
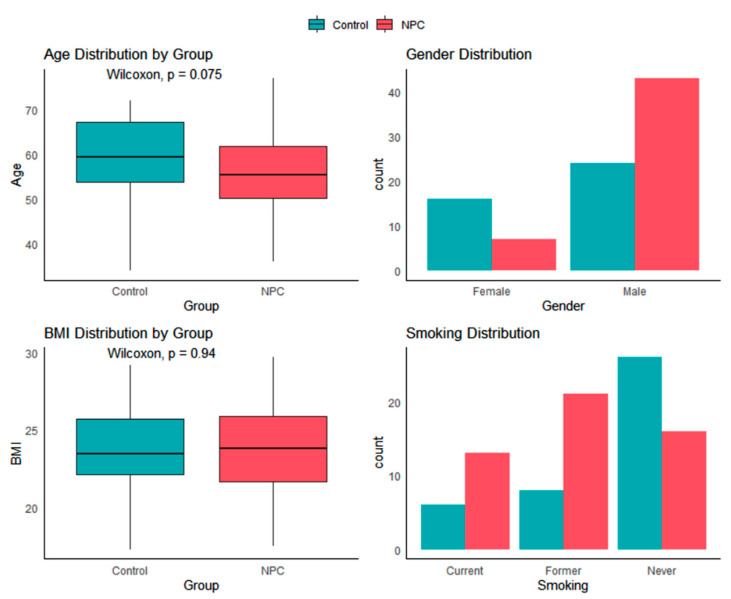
Baseline characteristics (age, gender, BMI, smoking) distribution in NPC and control groups.

To visualize the distribution of potential confounding factors between the NPC and healthy control groups, we plotted continuous variables (age and BMI) using boxplots and categorical variables (gender and smoking status) using bar plots as seen in Figure 1. Statistical comparisons between the groups were performed for continuous variables using the stat_compare_means function from the ggpubr package, while categorical variables were displayed as counts for each group. The four plots were arranged in a 2 × 2 panel to provide a clear overview of the distributions of key baseline characteristics and to visually assess potential imbalances between the groups.

To assess whether potential confounding factors differed between the NPC and control groups as shown in Table 5, we performed statistical tests on each baseline characteristic. For continuous variables (age and BMI), the Wilcoxon rank-sum test was used due to the potential non-normality of the data. For categorical variables (gender and smoking status), the chi-squared test was performed to compare the distributions between groups. This analysis aimed to identify any significant imbalances in key baseline characteristics that could act as confounders in subsequent analyses.

Group comparison tests revealed significant differences in gender distribution (χ^2^ = 6.59, *p* = 0.01) and smoking status (χ^2^ = 9.80, *p* = 0.01) between the NPC patients and controls, while age showed a non-significant trend (*p* = 0.08). No group difference was observed for BMI (*p* = 0.94), suggesting balanced baseline characteristics for this variable.

#### 3.2.3. Effects of Confounding Factors on Multiple VOCs

A multivariate analysis of variance (MANOVA) was performed to evaluate the effects of potential confounding factors—including age, gender, BMI, medication use, and smoking status—on all measured VOCs under the assumption that the data followed a normal distribution. Pillai’s trace was used as the test statistic to assess the overall multivariate effect of each factor. The corresponding approximate F-values, degrees of freedom, and *p*-values were calculated and summarized in a table, providing a quantitative assessment of how these baseline characteristics influenced the VOC profile.

The MANOVA results as shown in Table 6 indicate that smoking status (Pillai’s trace = 0.46, *p* = 0.0036) and medication use (Pillai’s trace = 0.27, *p* = 0.0065) significantly influence the VOC profiles, while BMI shows a marginal effect (*p* = 0.0358). Age and gender demonstrate no statistically significant associations (*p* > 0.05).

#### 3.2.4. Principal Component Analysis (PCA)

**Figure 2:**
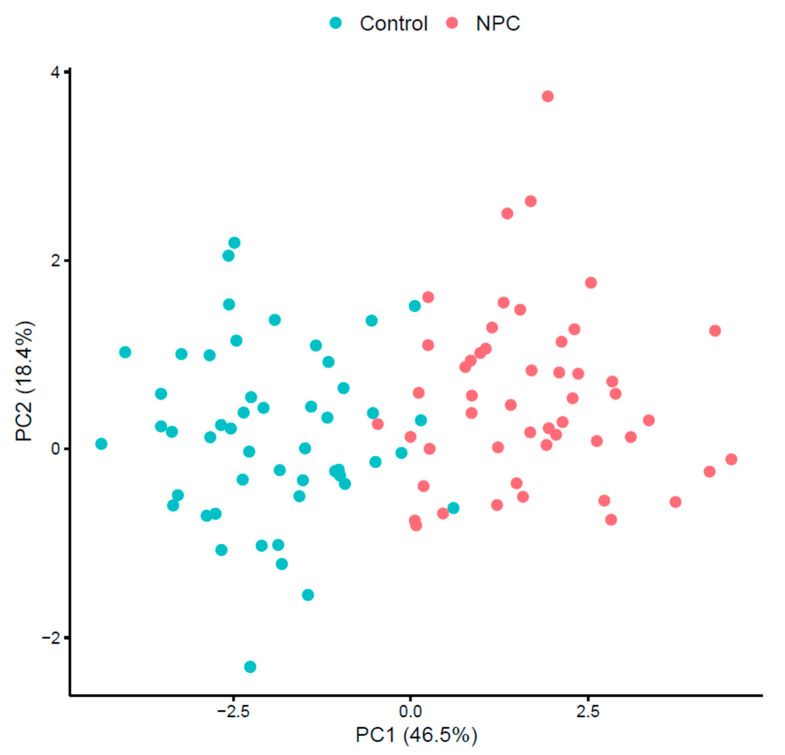
PCA plot for NPC vs. control.

The PCA plot (Figure 2) for the NPC and control subjects shows distinct clustering. This separation underscores the differences in VOC profiles between the NPC patients and healthy individuals. The first two principal components again accounted for a large portion of the variance, demonstrating the discriminatory power of the identified VOCs.

**Figure 3:**
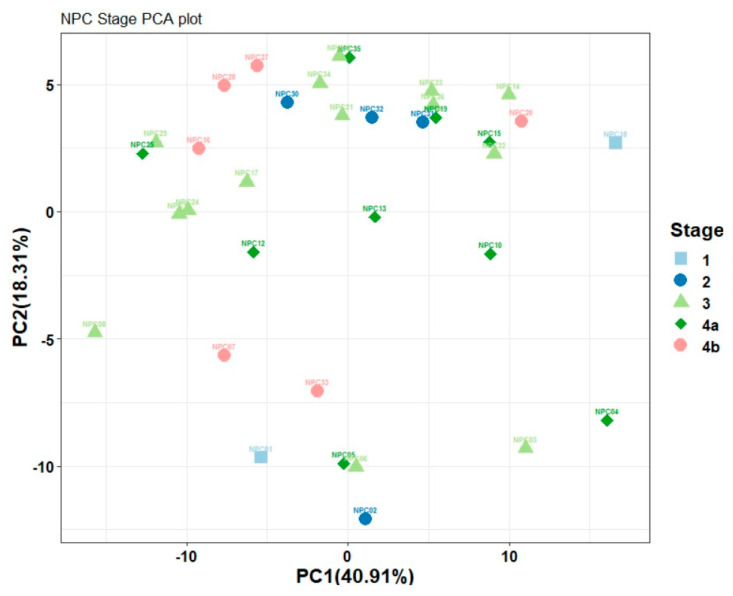
PCA plot for NPC stages.

The principal component analysis (PCA) plot in Figure 3 demonstrates the distribution of nasopharyngeal carcinoma (NPC) patients across different stages based on their volatile organic compounds (VOCs). While the variance captured by PC1 and PC2 is substantial, the overall separation between disease stages is not distinct. There is overlap, especially between the early stages (1 and 2) and more advanced stages (3, 4a, and 4b), indicating a lack of significant differentiation in the VOC profiles between these groups.

The clustering observed does suggest some variation as the disease progresses, particularly between the early and advanced stages. However, the data lacks statistical significance, with considerable overlap and heterogeneity within stages. This underscores the need for further analysis and larger datasets to identify reliable biomarkers for NPC staging through VOC profiling.

**Figure 4:**
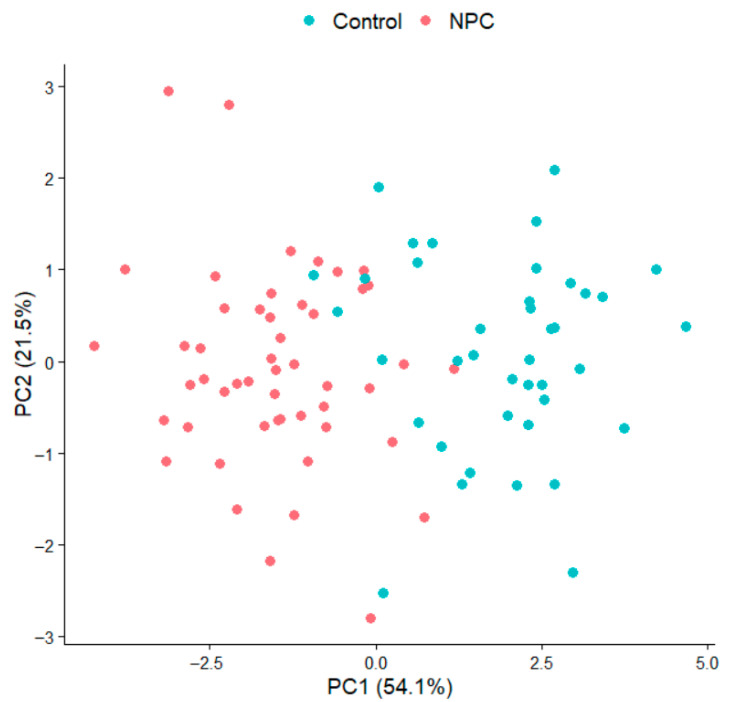
PCA after adjusting for age, gender, BMI, medication, and smoking.

To eliminate the influence of confounding factors such as age, gender, BMI, medication use, and smoking status on the analysis of volatile organic compounds (VOCs) and to more clearly identify nasopharyngeal carcinoma (NPC)-specific metabolic signatures, this study employed a confounder-adjusted principal component analysis (PCA) approach. First, we calculated the residuals for each VOC using a linear regression model (VOC ~ age + gender + BMI + medication + smoking) to remove the variance explained by these covariates. Subsequently, PCA was performed on the residual matrix. The results showed that the first two principal components (PC1 and PC2) of the adjusted data explained 54.1% and 21.5% of the variance, respectively, and the NPC patients and healthy controls exhibited clear separation in the PC1-PC2 space (see Figure 4). This indicates that after controlling for confounders, the disease-specific VOC patterns were significantly enhanced.

#### 3.2.5. Heatmap Analysis

**Figure 5:**
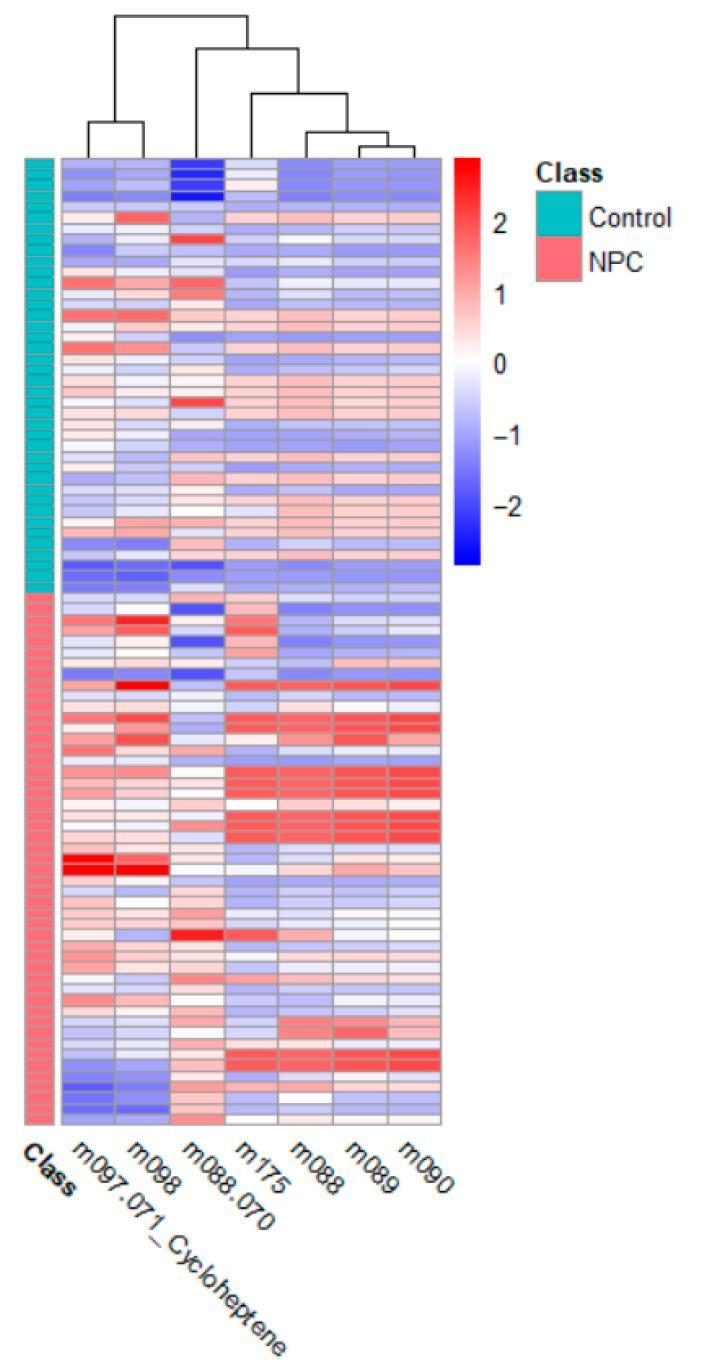
Heatmap for NPC vs. control.

Figure 5 presents the heatmap for the NPC and control groups. The heatmap reveals specific VOCs with elevated concentrations in the NPC patients, consistent with their role in tumor metabolism. The X-axis represents the VOCs, while the Y-axis corresponds to the individual samples. The color gradient, ranging from −2 to +2, reflects normalized concentrations, where positive values indicate higher concentrations and negative values represent lower concentrations relative to the overall dataset. The distinct clustering of the NPC patients further validates the identified VOCs as potential biomarkers.

#### 3.2.6. Correlation Map

**Figure 6:**
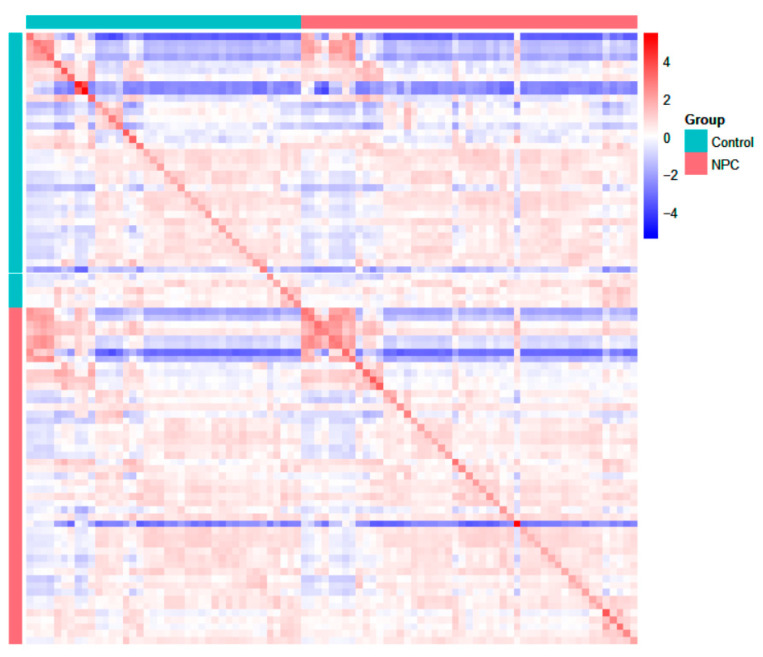
Correlation map for NPC and healthy controls.

The correlation map (Figure 6) for the NPC patients and healthy controls illustrates the relationships between the participants’ overall VOC profiles. Each cell in the matrix represents the correlation between the two participant groups, calculated based on the full group of VOC concentrations. Red shades indicate a positive correlation, meaning that the participants within the same group (NPC or healthy controls) have similar VOC concentration patterns. In contrast, blue shades indicate a negative correlation, where the participants from the different groups (NPC vs. healthy controls) show divergent VOC profiles. The map shows clusters of positive correlations within the NPC group and within the healthy control group, while negative correlations are seen between NPC patients and healthy controls. These patterns suggest that there are consistent metabolic differences between the two groups, offering insights into VOCs that may serve as potential biomarkers for NPC diagnosis. Further analysis could help identify specific VOCs driving these correlations, which may support the development of non-invasive diagnostic tools.

#### 3.2.7. Model Performance

**Figure 7:**
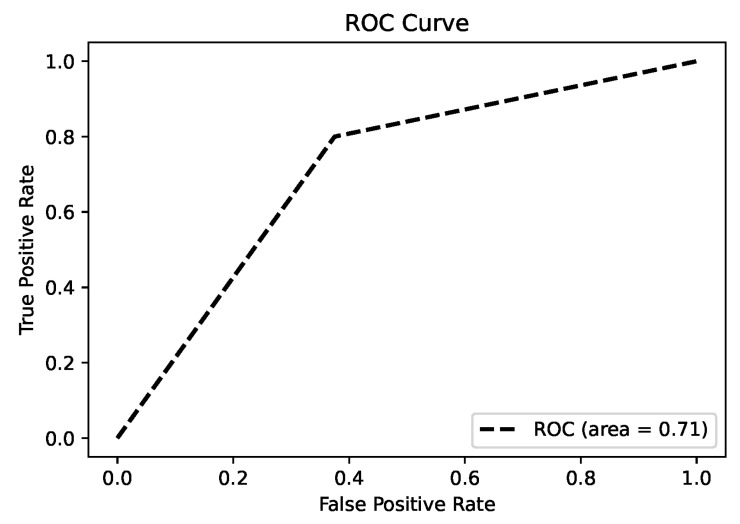
ROC curve of the random forest model for NPC vs. control classification (AUC = 0.71).

**Figure 8:**
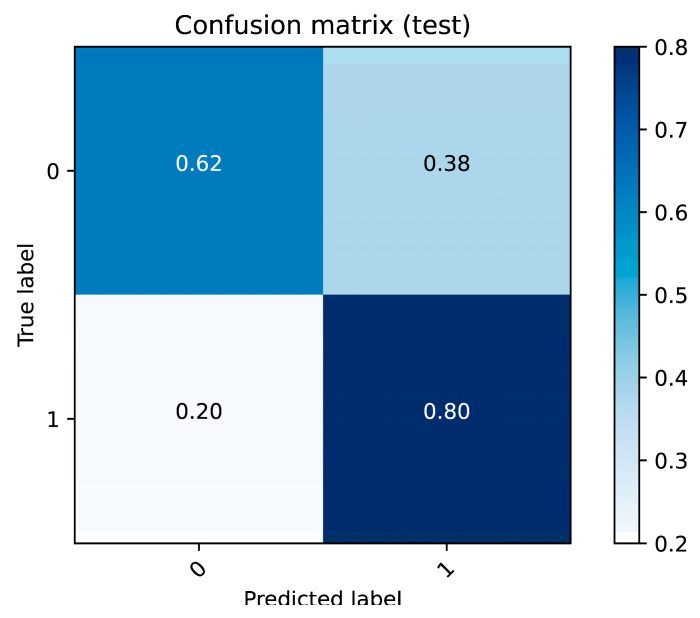
Confusion matrix of the random forest model.

The random forest model was employed to distinguish between NPC patients and healthy controls. The receiver operating characteristic (ROC) curve, as shown in Figure 7, achieved an area under the curve (AUC) of 0.71, indicating moderate classification performance. The confusion matrix in Figure 8 provides further insights into the model’s performance, showing that the model correctly classified 62% of the true negative cases and 80% of the true positive cases. However, the false-positive rate was 38% and the false-negative rate was 20%, suggesting that while the model performs relatively well in identifying NPC cases, improvements are needed to reduce false positives and enhance overall prediction accuracy.

## 4. Discussion

The results of this study underscore the potential of proton transfer reaction mass spectrometry (PTR-MS) in identifying volatile organic compound (VOC) biomarkers for diagnosing nasopharyngeal carcinoma (NPC). Significant differences in VOC profiles between the NPC patients and control subjects were observed, identifying specific biomarkers that can effectively distinguish between diseased and healthy individuals.

In the discussion of the potential identities of m089 and m175, it is essential to hypothesize the possible compounds based on their mass-to-charge (*m*/*z*) ratios and their relevance to nasopharyngeal carcinoma (NPC). Given that PTR-MS assigns a VOC ID that is one unit larger than the molecular weight due to proton attachment, these *m*/*z* values can provide insights into their molecular identities and their roles in NPC.

M089 and m175 likely represent VOCs that are products of altered metabolic processes in NPC. These compounds may serve as biomarkers for the disease, reflecting the oxidative stress and lipid metabolism disruptions that are critical in the progression of NPC. Further research is required to confirm the exact identities of these VOCs and elucidate their roles in NPC pathophysiology, but their significant presence suggests that they could be valuable for early diagnosis and monitoring.

M089 likely corresponds to a molecule with a molecular weight of approximately 88 Da, with butyric acid as a potential candidate. Butyric acid is a short-chain fatty acid involved in essential metabolic processes, including inflammation and immune regulation [24]. Elevated levels may indicate dysregulated fatty acid metabolism, a phenomenon frequently associated with cancer. Ji et al. (2025) showed that the exhaled breath profiles of colorectal cancer patients exhibited significant alterations in VOCs related to short-chain fatty acid metabolism, including butyric acid [25], underscoring the role of short-chain fatty acid dysregulation in malignancy. In the context of NPC, butyric acid may therefore serve as a marker of altered energy metabolism or an inflammatory response associated with tumor progression.

Regarding m175, we were unable to conclusively determine the corresponding compound. The *m*/*z* value of 175 represented a wide range of molecules, possibly related to fatty acid derivatives or other metabolic byproducts. However, without a clear chemical identification, it remains speculative. Further analysis, such as detailed mass spectrometry or database matching, is needed to accurately characterize m175 and assess its potential relevance in NPC. Identifying this compound could provide more insight into the metabolic alterations occurring in NPC patients.

A key contribution of this research is its novel application of PTR-MS for the non-invasive detection of NPC. Traditional diagnostic methods, such as nasopharyngoscopy for NPC, are invasive, time-consuming, and costly. In contrast, PTR-MS breath analysis offers a rapid, non-invasive, and cost-effective alternative suitable for clinical settings. Detection of significant VOC features, such as *m*/*z* 89 and *m*/*z* 175 in NPC patients, highlights the potential sensitivity and specificity of this method [26].

This study also enhances the understanding of the metabolic changes associated with NPC. The correlation analyses reveal potential metabolic pathways involved in this disease, providing insights for future research and therapeutic strategies. The identified VOCs associated with NPC could inform the development of targeted therapies and improve patient outcomes.

Improvements over previous studies include a comprehensive analysis of multiple VOCs and the application of advanced statistical techniques to validate the findings. Principal component analysis (PCA), heat maps, and correlation maps provide a robust framework for visualizing and interpreting the data, enhancing the reliability of the results.

The demographics of our NPC group closely resemble those of other studies, with a mean age at diagnosis ranging from 55 to 60 years [27,28], and over 70% of patients being diagnosed at advanced stages (Stage III and IV) [27]. Although there are notable differences in gender and age between the NPC and control groups, other studies have indicated that variations in exhaled breath VOC content are not significantly affected by these factors [29,30]. Although most patients in our cohort had advanced-stage NPC, consistent with regional epidemiology, the scientific and clinical value of PTR-MS breath analysis lies in its potential to detect NPC at earlier stages (I/II). Early detection is critical for improving survival, and PTR-MS offers a non-invasive screening approach that could complement existing diagnostic pathways. Larger future studies with sufficient early-stage patients are needed to validate this application.

The identification of VOCs with consistent diagnostic value across different populations is essential for the clinical translation of PTR-MS breath analysis [31]. In the context of NPC, our findings highlight m089 and m175 as promising candidates, but validation in larger and more diverse cohorts is needed to confirm their reliability. Future studies should also investigate the integration of VOC profiling with established diagnostic modalities such as EBV serology and nasoendoscopy to enhance accuracy and improve patient outcomes. In addition, evaluating the temporal stability of these biomarkers and their response to treatment could provide important insights into NPC progression and therapeutic efficacy [23,31].

A limitation of this study is that the putative biomarkers m089 and m175 were not chemically confirmed. In future work, we plan to analyze pure standard compounds and compare their PTR-MS spectra with breath measurements to definitively establish compound identities.

In summary, this study demonstrates the feasibility and potential of PTR-MS for the non-invasive screening of NPC. This approach offers a promising tool for the early detection and monitoring of this condition, with significant implications for clinical practice and patient care.

## 5. Conclusions

This study demonstrates that PTR-MS breath analysis has potential scientific and clinical utility as a non-invasive screening tool for the early detection of NPC. By identifying disease-specific VOCs such as m089 and m175, this technique offers a rapid and patient-friendly alternative that could complement existing diagnostic pathways, reduce reliance on invasive procedures, and improve accessibility of screening in community settings. Future validation in larger and more diverse cohorts is warranted to establish its role in clinical practice.

## Figures and Tables

**Table 1:** Technical specifications of PTR-MS.

Parameter	Specification
Mass resolution	>1500 m/Δm (FWHM) for *m/z* > 79
Response time	<100 ms
Sensitivity	>200 cps/ppbv for *m/z* 181
Detection limits	<10 pptv for *m/z* 181 (averaged over 1 min)
Linearity range	10 pptv–1 ppmv
Adjustable flow	50–800 sccm

**Table 2:** Study population for NPC and control groups.

	NPC	Control
Number	50	40
Age (mean ± SD)	58.98 ± 11.38	43.38 ± 12.70
Gender M/F (%)	43/7 (86.0%/14.0%)	24/16 (60.0%/40.0%)
Stage I (%)	3 (6.0%)	-
Stage II (%)	8 (16.0%)	-
Stage III (%)	18 (36.0%)	-
Stage IVa (%)	15 (30.0%)	-
Stage IVb (%)	6 (12.0%)	-

**Table 3:** Biomarkers identified for NPC with their corresponding *p*-values.

Biomarker (*m*/*z*)	*p*-Value
m088	0.019
m088.070	0.041
m089	0.006
m090	0.006
m097.071 Cycloheptene	0.021
m098	0.019
m175	0.001

**Table 4:** Baseline characteristics and group balance between NPC and healthy controls.

	Level	Control	NPC	*p*_Value	Test SMD
n		40	50		
Age (mean (SD))		43.38 ± 12.70	58.98 ± 11.38	0.117	0.335
Gender	Female	16 (40.0)	7 (14.0)	0.010	0.612
	Male	24 (60.0)	43 (86.0)		
BMI (mean (SD))		23.67 ± 2.74	23.81 ± 2.87	0.816	0.050
Medication (%)	No	29 (72.5)	37 (74.0)	1.000	0.034
	Yes	11 (27.5)	13 (26.0)		
Smoking (%)	Current	6 (15.0)	13 (26.0)	0.007	0.703
	Former	8 (20.0)	21 (42.0)		
	Never	26 (65.0)	16 (32.0)		

**Table 5:** Baseline confounder comparison between NPC and control groups.

Variable	Test	Statistic	df	*p*_Value
Age	Wilcoxon rank-sum	1219.50	NA	0.08
BMI	Wilcoxon rank-sum	991.00	NA	0.94
Gender	Chi-squared	6.59	1	0.01
Smoking	Chi-squared	9.80	2	0.01

**Table 6:** Impact of baseline confounders on VOCs.

	Df	Pillai	Approx_F	Den_Df	*p*_Value	Significance
Age	1	0.10	0.79	74	0.6346	
Gender	1	0.19	1.77	74	0.0804	
BMI	1	0.22	2.09	74	0.0358	*
medication	1	0.27	2.73	74	0.0065	**
Smoking	2	0.46	2.22	150	0.0036	**
Residuals	83					

** Indicates statistical significance, while * indicates marginal significance.

## Data Availability

The original contributions presented in the study are included in the article, further inquiries can be directed to the corresponding author.

## Abbreviations

The following abbreviations are used in this manuscript:

NPC	Nasopharyngeal Carcinoma
PTR-MS	Proton Transfer Reaction Mass Spectrometry
VOC	Volatile Organic Compound
EBV	Epstein–Barr Virus
MRI	Magnetic Resonance Imaging
CT	Computed Tomography
AJCC	American Joint Committee on Cancer
TOF-MS	Time-of-Flight Mass Spectrometer
